# Supplementary material for: Characteristics of patients with longer treatment period of lenvatinib for unresectable hepatocellular carcinoma: A post-hoc analysis of post-marketing surveillance study in Japan
Source: PLoS One. 2024 Mar 8;19(3):e0298420. doi: 10.1371/journal.pone.0298420 (PMC10923456; doi:10.1371/journal.pone.0298420)
Supplement: S1 Table — aThe proportion of ADRs ≥1% are presented in the table. ADR, adverse drug reaction; HFSR, hand-foot skin reaction. (DOCX) [file pone.0298420.s001.docx]

**S1 Table. The occurrence of ADRs^a^ that lead to treatment discontinuation (n = 703)**

| **ADR, n (%)** | **Any grade** |
| --- | --- |
| Appetite loss | 44 (6.3) |
| Fatigue | 33 (4.7) |
| Proteinuria | 23 (3.3) |
| Hepatic encephalopathy | 17 (2.4) |
| Diarrhea | 13 (1.8) |
| HFSR | 10 (1.4) |
| Nausea | 9 (1.3) |
| Ascites | 8 (1.1) |
| Hepatic dysfunction | 8 (1.1) |
| Hypothyroidism | 7 (1.0) |

^a^The proportion of ADRs ≥1% are presented in the table.

ADR, adverse drug reaction; HFSR, hand-foot skin reaction.
