# Supplementary material for: Characteristics of patients with longer treatment period of lenvatinib for unresectable hepatocellular carcinoma: A post-hoc analysis of post-marketing surveillance study in Japan
Source: PLoS One. 2024 Mar 8;19(3):e0298420. doi: 10.1371/journal.pone.0298420 (PMC10923456; doi:10.1371/journal.pone.0298420)
Supplement: S2 Table — aOf 703 patients, 550 patients had ≥8 weeks of treatment. Of these, 170 patients had no dose modification and 380 had dose modification, during the initial eight weeks of treatment. Of these, 126 and 297 patients (423 in total) with tumor assessment data using the mRECIST criteria were analyzed. bFisher’s exact test, cORR = proportion of patients with CR and PR, dDCR = proportion of patients with CR, PR, and SD, CR, complete response; DCR, disease control rate; mRECIST, modified Response Evaluation Criteria in Solid Tumors; ORR, objective response rate; PD, progressive disease; PR, partial response; RDI, relative dose intensity; SD, stable disease. (DOCX) [file pone.0298420.s002.docx]

**S2 Table. Tumor response by with or without dose modification during the initial eight weeks of treatment in patients with ≥8 weeks of treatment (n = 423^a^)**

| Response rate, n (%) | No dose modification  (8-week RDI ≥100%) | Dose modification  (8-week RDI <100%) | p-value^b^ |
| --- | --- | --- | --- |
|  | (n = 126) | (n = 297) |  |
| ORR^c^ | 66 (52.4) | 118 (39.7) | 0.018 |
| DCR^d^ | 115 (91.3) | 246 (82.8) | 0.024 |
| Best overall response |  |  |  |
| CR | 8 (6.3) | 23 (7.7) | 0.687 |
| PR | 58 (46.0) | 95 (32.0) | 0.007 |
| SD | 49 (38.9) | 128 (43.1) | 0.451 |
| PD | 11 (8.7) | 51 (17.2) | 0.024 |

^a^Of 703 patients, 550 patients had ≥8 weeks of treatment. Of these, 170 patients had no dose modification and 380 had dose modification, during the initial eight weeks of treatment. Of these, 126 and 297 patients (423 in total) with tumor assessment data using the mRECIST criteria were analyzed.

^b^Fisher’s exact test

^c^ORR = proportion of patients with CR and PR

^d^DCR = proportion of patients with CR, PR, and SD

CR, complete response; DCR, disease control rate; mRECIST, modified Response Evaluation Criteria in Solid Tumors; ORR, objective response rate; PD, progressive disease; PR, partial response; RDI, relative dose intensity; SD, stable disease.
