# Supplementary material for: Characteristics of patients with longer treatment period of lenvatinib for unresectable hepatocellular carcinoma: A post-hoc analysis of post-marketing surveillance study in Japan
Source: PLoS One. 2024 Mar 8;19(3):e0298420. doi: 10.1371/journal.pone.0298420 (PMC10923456; doi:10.1371/journal.pone.0298420)
Supplement: S1 Fig — (a) Treatment duration was significantly longer in patients with objective response (CR or PR) than in those without an objective response (SD or PD). (b) Overall survival was significantly longer in patients with objective response (CR or PR) than in those without an objective response (SD or PD). CR, complete response; PD, progressive disease; PR, partial response; SD, stable disease. (DOCX) [file pone.0298420.s003.docx]

**S1 Fig. Kaplan-Meier curves for (a) treatment duration and (b) overall survival according to treatment response (CR or PR vs. SD or PD)**


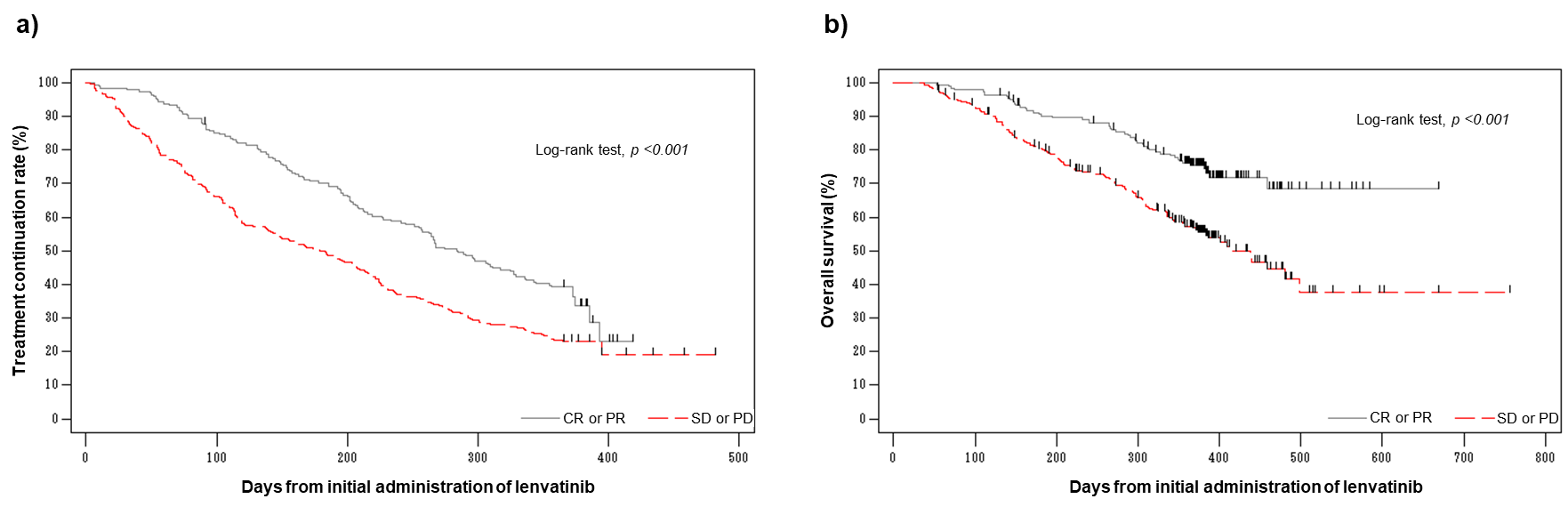


1. Treatment duration was significantly longer in patients with objective response (CR or PR) than in those without an objective response (SD or PD).
2. Overall survival was significantly longer in patients with objective response (CR or PR) than in those without an objective response (SD or PD).

CR, complete response; PD, progressive disease; PR, partial response; SD, stable disease.
