# Supplementary material for: Characteristics of patients with longer treatment period of lenvatinib for unresectable hepatocellular carcinoma: A post-hoc analysis of post-marketing surveillance study in Japan
Source: PLoS One. 2024 Mar 8;19(3):e0298420. doi: 10.1371/journal.pone.0298420 (PMC10923456; doi:10.1371/journal.pone.0298420)
Supplement: S2 Fig — The RDI was calculated as the ratio of the total dosage delivered to the body weight-based standard dosage (i.e., the treatment duration [day] multiplied by 12 mg or 8 mg, depending on body weight). The mean RDI was around 80% initially, then gradually declined to approximately 60% in a few months, and sustained at the level after that. RDI, relative dose intensity. (DOCX) [file pone.0298420.s004.docx]

**S2 Fig. The transition of mean RDIs per two weeks in the overall study population (n = 703)**


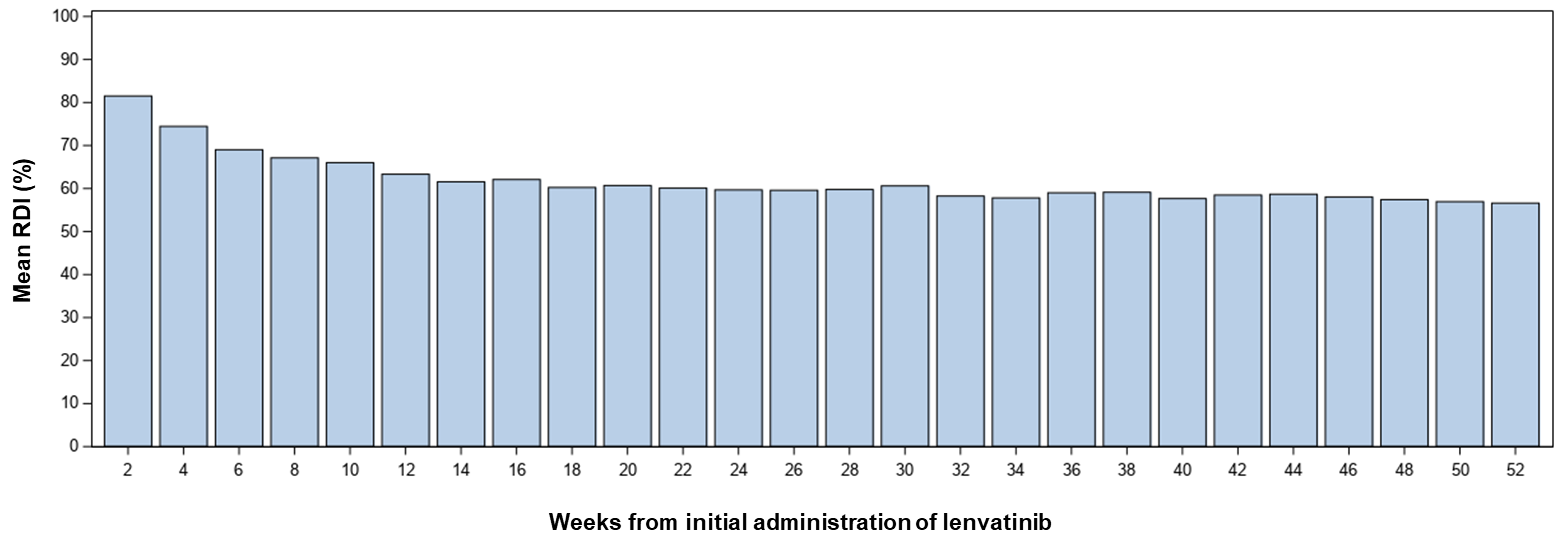


The RDI was calculated as the ratio of the total dosage delivered to the body weight-based standard dosage (i.e., the treatment duration [day] multiplied by 12 mg or 8 mg, depending on body weight). The mean RDI was around 80% initially, then gradually declined to approximately 60% in a few months, and sustained at the level after that.

RDI, relative dose intensity.
