# Supplementary material for: Characteristics of patients with longer treatment period of lenvatinib for unresectable hepatocellular carcinoma: A post-hoc analysis of post-marketing surveillance study in Japan
Source: PLoS One. 2024 Mar 8;19(3):e0298420. doi: 10.1371/journal.pone.0298420 (PMC10923456; doi:10.1371/journal.pone.0298420)
Supplement: Attachment — Submitted filename: ResponseLetter_231206_231208.docx [file pone.0298420.s005.docx]

December 6, 2023

Editorial Operations Managers

*PLOS ONE*

Jin-Yu Sun

Academic Editor

*PLOS ONE*

Dear Dr. Sun and Editorial Team:

My coauthors and I wish to submit the attached revised manuscript titled “Characteristics of patients with longer treatment period of lenvatinib for unresectable hepatocellular carcinoma: A post-hoc analysis of post-marketing surveillance study in Japan” for publication in *PLOS ONE*.

We greatly appreciate valuable comments and editorial supports provided by the two reviewers and Editorial Team of *PLOS ONE*. We have amended our manuscript according to the comments provided by reviewers wherever possible. We believe that their comments have enabled us to improve the content of the manuscript. Please find our responses to reviewers’ comments.

As per journal requirements, we have amended Competing Interest Statement and Data Availability Statement. The amended statements are also provided below. No amendments were made for Financial Disclosure but is included in this cover letter. Additionally, no amendments were made for Ethics Statement in the “Methods” section. Thank you very much in advance to amend these statements on our behalf.

**Competing Interest Statement**

I have read the journal's policy and the authors of this manuscript have the following competing interests:

Tatsuya Yamashita has received travel grants and honoraria for speaking or participation at meetings from Chugai Pharma, Eli Lilly Japan, and Eisai.

Natsumi Suzuki, Katsuaki Motoyoshi, and Wanjun Zhu are employees of Eisai Co., Ltd.

Junji Furuse received research grants from Astellas, Astra Zeneca, Incyte Biosciences Japan, Eisai, MSD, Ono Pharmaceutical, Sanofi, J-Pharma, Daiichi Sankyo, Sumitomo Dainippon, Taiho Pharmaceutical, Takeda, Delta-Fly-Pharma, Chugai Pharma. He has received travel grants and honoraria for speaking or participation at meetings from Ono Pharmaceutical, Chugai Pharma, Incyte Biosciences Japan, Eisai, Eli Lilly Japan, Astra Zeneca, Yakult Honsha, Servier Japan, MSD, Novartis Pharma, Takeda, Bayer, Taiho Pharmaceutical, EA Pharma, Teijin pharma, Daiichi Sankyo, Terumo, Fuji film, Astellas, Onco Therapy Science, Delta-Fly-Pharma, Merck Bio, Incyte Biosciences Japan, J-Pharma.

This does not alter our adherence to PLOS ONE policies on sharing data and materials.

**Data Availability Statement**

Individual data were provided to Eisai under a contract between the participating institutions and Eisai. Due to restrictions by Eisai, the underlying data may not be made publicly available. However, data are available to interested and qualified researchers who meet the criteria for access to confidential data upon request to the Clinical Trial Disclosure site of Eisai. For further information, please access the following link: https://www.eisai.co.jp/innovation/research/clinical_trials/clinical/index.html (Japanese); https://www.eisai.com/innovation/research/clinical_trials/clinical/index.html?_gl=1*801ekg*_ga*MjI5NDMwMjY3LjE2OTgzNjcwNzE.*_ga_X1FWS6YR87*MTY5ODM2NzA3MS4xLjAuMTY5ODM2NzA3OC41My4wLjA (English).

**Financial Disclosure (Not Amended)**

This study was funded by Eisai Co., Ltd. The sponsor was involved in the study design, data collection and analysis, decision to publish, and preparation of the manuscript.

Lastly, we believe that the findings of this study are relevant to the scope of your journal and will be of interest to your readership.

Thank you for your consideration. I look forward to hearing from you.

Sincerely yours,

Tatsuya Yamashita

Department of Gastroenterology, Kanazawa University Hospital, Kanazawa, Japan

ytatsuya28@gmail.com

Reviewer 1

| Comments | Responses and revisions |
| --- | --- |
| This article by Yamashita et al. analyzes real-world clinical data of lenvatinib treatment in patients with unresectable hepatocellular carcinoma. The authors focused on longer lenvatinib treatment group and compared characteristics with those of shorter treatment. They also analyzed the impact of dose modification on the efficacy and adverse events in longer treatment group. The reviewer believes that this paper provides important real-world data that will benefit clinicians.  Please consider the following point. | ‐ |
| #1 the authors did not perform statistical analyses of the longer vs. shorter treatment and dose-modified vs. non-modified groups. The reviewer believe that it is a rigorous method not to make statistical comparisons in post-hoc analyses. However, there are several points where statistical comparisons can be made, such as comparing any ADR or radiological response between patients experienced dose modification within 177 days and those did not. Please comment on this point. | [Response]  Your feedback is greatly valued, and we fully understand the importance of including a statistical analysis. In our initial work, we did not carry out a statistical examination due to concerns regarding selection bias among subgroups. However, in the revised version, we have incorporated a thorough statistical analysis into our study. Please kindly examine the Figures and Tables, to gauge the impact of these improvements on our findings. |

Reviewer 2

| Comments | Responses and revisions |
| --- | --- |
| T. Yamashita et al. have reported about a post-hoc analysis of post-marketing surveillance study in Japan. To establish the position of LEN in the present guidelines, it is also very important to investigate about post-hoc analysis about LEN in Japanese population. However, there are some concerns in this study to be published as an original article. | ‐ |
| #1 . There is no data provided for the statistical analysis in the document and Tables. It would be helpful if the authors could explain the reason for this omission. | [Response]  Your feedback is greatly valued, and we fully understand the importance of including a statistical analysis. In our initial work, we did not carry out a statistical examination due to concerns regarding selection bias among subgroups. However, in the revised version, we have incorporated a thorough statistical analysis into our study. Please kindly examine the Figures and Tables, to gauge the impact of these improvements on our findings. |
| #2. If possible, authors may add the data about the reason why LEN was discontinued. Is it progressive disease, discontinuation due to ADR, or post-LEN therapy (e.g. TACE) ? | [Response]  We appreciate your comment. We have included the reason for the discontinuation in the manuscript, and we kindly request that you review it for further details (Line 359–362 in the Revised manuscript with track changes). |
| #3. While the median treatment duration is mentioned for the entire cohort, it would be beneficial to provide the minimum and maximum values for treatment duration within both longer and shorter treatment groups, respectively. | [Response]  We appreciate your suggestion. We have now incorporated the minimum and maximum values (ranging from 2 days to 482 days) into the text, and those in 2 cohorts (Longer: range 177–482 days, Shorter: range 2–174 days) and we kindly ask you to verify this inclusion (Line 168 and Line 171–172). |
| #4. It's noted that the Relative Dose Intensity (RDI) cut-off is set at 100%. Have the 　authors explored other RDI cut-off values? | [Response]  We appreciate your comments, and we've given careful consideration to this matter. With respect to the cutoff value, it is noteworthy that previous reports commonly mention an 8-week RDI. However, there remains uncertainty about which specific time point for RDI is most appropriate or whether RDI is a suitable indicator, given the limited evidence available. In light of this context, our post-hoc analysis examined the results by categorizing them into two groups: "Full dose" and "Dose modification," using the median administration duration as the time point. |
| #5. Is there any available data regarding the post-LEN treatment administered to the patients in the study ? | [Response]  Thank you for the confirmation.  Since this PMS was designed to assess the safety and dosing of lenvatinib, we did not collect information on post-treatment. |
| #6. Have the authors investigated the longitudinal changes of hepatic function such as Child-Pugh, ALBI, etc. at different time points (e.g., pre-treatment, 4 weeks, 8 weeks, and 12 weeks)? Which show differences in longer vs. shorter? I am interested in whether liver function of longer-treatment patients to be better maintained. | [Response]  Thank you for the confirmation.  Since this PMS was designed to assess the safety and dosing of lenvatinib, we did not collect information on the longitudinal changes of hepatic function. |
